# Osteocalcin associates with bone mineral density and *VDR* gene polymorphisms in type 1 and type 2 diabetes

**DOI:** 10.1515/almed-2023-0131

**Published:** 2023-10-24

**Authors:** Carla Ramírez Ruiz, Nerea Varo Cenarruzabeitia, Miriam Martínez Villanueva, Antonio M. Hernández Martínez, José Antonio Noguera Velasco

**Affiliations:** Department of Clinical Biochemistry, Clínica Universidad de Navarra, Madrid, Spain; Department of Clinical Biochemistry, Clínica Universidad de Navarra, Pamplona, Spain; Department of Clinical Biochemistry, Hospital Clínico Universitario Virgen de la Arrixaca, Murcia, Spain; Endocrinology and Nutrition Department, Hospital Clínico Universitario Virgen de la Arrixaca, Murcia, Spain; Servicio de Bioquímica, Clínica Universidad de Navarra – Madrid, Madrid, Spain

**Keywords:** bone, bone turnover markers, diabetes mellitus, osteocalcin, osteoporosis, *VDR* polymorphisms

## Abstract

**Objectives:**

Bone metabolism is impaired in diabetes mellitus (DM). Our objective is to evaluate the association of bone turnover markers (BTM) and vitamin D receptor (*VDR*) gene polymorphisms with bone mineral density (BMD) in DM type 1 (T1D) and DM type 2 (T2D).

**Methods:**

A total of 165 patients (53 T1D and 112 T2D) were enrolled. BMD was measured by dual-energy X-ray absorptiometry (DEXA). Plasma osteocalcin (OC), beta-CrossLaps (β-CTX) and N‐amino terminal propeptide of type I collagen (P1NP) and *VDR* gene polymorphisms were evaluated.

**Results:**

Participants were 53 T1D (41 years [31–48]) and 112 T2D (60 years [51–66]). BMD were not statistically different between the groups. OC (p<0.001) and P1NP levels (p<0.001) were higher in patients with T1D. The areas under the curve for the prediction of bone pathology were 0.732 (p=0.038) for OC in T1D and 0.697 (p=0.007) in T2D. A significant association was found between lower lumbar BMD and the A allele of *BsmI* (p=0.03), the A allele of *ApaI* (p=0.04) and the allele C of the *Taql* (p=0.046). Also, a significant correlation was found with higher OC levels and the G allele of *BsmI* (p=0.044), C allele of *ApaI* (p=0.011), T allele of *Taql* (p=0.006) and with C allele of *FokI* (p=0.004).

**Conclusions:**

The high negative predictive value of the cut-off point for OC suggests that could be useful in excluding the risk suffering bone loss, allowing offering a personalized clinical approach to prevent this pathology.

## Introduction

Diabetes mellitus (DM) is characterized by elevated blood glucose, which over time not also leads to serious damage to the heart, blood vessels and other organs, but also confers increased risk of bone fracture. Several mechanisms such as hyperglycemia, insulin, oxidative stress, vitamin D deficiency, etc. occur in both types of diabetes that can affect bone strength and metabolism [1]. Thus, it is reasonable to screen for osteoporosis in patients with diabetes.

The gold standard to diagnose osteoporosis or osteopenia according to the criteria of the World Health Organization (WHO) is the assessment of bone mineral density (BMD) [2]. However, it has some limitations as it does not provide information about bone quality. Also, the diagnosis of osteoporosis in diabetic patients is challenging as paradoxically, DM type 2 (T2D) tend to have adequate or high BMD but increased fracture risk [3].

For that reason, other diagnostic approaches are required to unravel the nexus between DM and osteoporosis. Bone turnover markers (BTM) have emerged as an alternative to BMD assessment, as they reflect the metabolic activity of bone. Though not validated for diagnosis, elevated levels of BTM predict bone loss and are adequate to assess the response to therapy.

Most of BTM studies have been performed in postmenopausal women or in older men. Studies evaluating the synthesis and resorption bone markers in DM type 1 (T1D) and T2D have obtained conflicting results as some have found decreased BTM levels or no differences [4], [5], [6]. It appears that diabetic patients show a dissociative pattern, as changes in formation are not mirrored by similar changes in resorption [7]. It is possible to find low BTM levels with low, normal or even elevated BMD in patient at increased risk of fracture suggesting that traditional reference ranges may not be adequate for clinical decisions [8]. So more studies are needed to establish the usefulness of BTM as a screening tool in diabetic population.

Also, as family and twin studies suggest that BMD has a high heritability [9], many studies have been performed to identify genes that contribute to the development and maintenance of bone mass. The vitamin D receptor gene (*VDR*) has been extensively studied due to the crucial role of vitamin D in bone metabolism [10, 11]. Genetic factors are also of great interest in diabetic patients but evidence is currently insufficient, vary among populations and limited studies combine *VDR* gene polymorphisms and BTM analysis [12, 13]. More knowledge is needed about the genetic contribution to bone metabolism in diabetes and factors that may identify patients who are at high risk of bone loss and therefore are more likely to require future management.

In this context, the objectives of the present study were: (1) to explore the association of levels of the BTM (osteocalcin [OC]), beta-CrossLaps [β-CTX] and N‐amino terminal propeptide of type I collagen [P1NP] with BMD in patients with T1D and T2D; (2) to provide information if genetic variants within the *VDR* gene polymorphisms may predispose to increased osteoporosis in diabetic patients.

## Materials and methods

### Subjects and clinical studies

We performed a prospective study in the Hospital Clínico Universitario Virgen de la Arrixaca (Murcia, Spain). The study population consisted of 165 Caucasian patients diagnosed of DM (53 type 1 and 112 with type 2) (age, 18–70 years) recruited at the Endocrinology Department.

Individuals with oncological disease, diabetes secondary to other pathologies and diseases or conditions affecting bone turnover were excluded from the study. Patients taking oral glucocorticoids, hormone replacement therapy or any treatment that could interfere with bone metabolism were also excluded.

At the time of enrolment, collected data on diabetes related complications, family history of fractures, smoking habits, alcohol use, physical activity, sun exposure, coffee consumption, diabetes duration and treatment. Height and weight were measured and body mass index (BMI) was calculated. Microvascular (nephropathy, retinopathy and neuropathy) and macrovascular (myocardial infarction, stroke and peripheral vascular disease) complications were compiled.

All subjects recruited provided written informed consent. The study was conducted in accordance with the Declaration of Helsinki, and the protocol was approved by the local Ethics Committee.

### Densitometry

BMD measurement was performed by dual-energy X-ray absorptiometry (DEXA) at the lumbar spine (L1–L4) and the three sites of the right hip (femoral neck) using Lunar DEXA device, DPX-L. Osteopenia was defined according to the WHO criteria, as T-score for the lumbar spine or femoral neck between −1 SD and −2.5 SD. Osteoporosis was defined as T-score at the lumbar spine or femoral neck lower than or equal to −2.5 SD for postmenopausal women and men over 50 years. For premenopausal women and men aged below 50 years, osteoporosis was diagnosed with a BMD Z-score of equal or less than −2 SD at the lumbar spine or femoral neck [14].

### Biochemical analyses

To avoid diurnal variation, blood samples were collected after overnight fasting between 8:00 and 10:00 into vacutainer tubes and an aliquot stored frozen at −80 °C until analysis. Measurement of OC, β‐CTX and P1NP was performed at the biochemistry laboratory of the Hospital Clínico Universitario Virgen de la Arrixaca by chemiluminescence (ECLIA) in a Cobas E411 analyzer (Roche Diagnostics). Intra‐ and interassay coefficients of variation were below 5 %. The detection limits were 0.50 μg/L for OC, 0.01 μg/L for β-CTX and 5 μg/L for P1NP.

### Polymorphisms

Four single nucleotide polymorphism (SNP) rs1544410, rs7975232, rs731236 and rs2228570 were selected tagging some of the most studied polymorphisms, namely, *BsmI*, *ApaI*, *TaqI* and *FokI*. Genotypic and allelic frequencies were compared with reference population (population European of 1000 Genomes Project and its subpopulation IBS [Iberian population in Spain]), data retrieved from ensembl.org.

Genomic DNA was isolated using the CLART^®^MetaBone extraction kit (GENOMICA) and stored at −20 °C until analysis. Genotyping was performed by DNA detection by HybProbe during real-time polymerase chain reaction on the LightCycler2.0^®^ (Roche Diagnostics^®^).

### Statistical analysis

Statistical analysis was performed using the IBM SPSS Statistics 23.0 software (IBM, New York, NY, USA). Normal distribution of samples was assessed by the Shapiro-Wilk test. Differences between study groups were evaluated by the Student’s t-test for normally or Mann-Whitney-U test for non-normally distributed variables, and chi square statistic for categorical variables. Pearson and Spearman’s correlation tests assessed univariate correlations. Multivariate linear regression analysis was performed to assess the independent relationships of BTM. ROC curve analysis was performed to determine the diagnostic performance of BTM. Youden’s index was used to determine an optimal cut-off value for the detection of bone pathology using BTM.

Data were summarized as mean ± Standard Error of the Mean (SEM) for quantitative variables and as frequencies for qualitative variables. Genotype distribution was tested for Hardy–Weinberg equilibrium using the chi-square test. The effect of genotypes on BMD was evaluated by variance analysis for repeated measurements. Statistical significance was established at p<0.05.

## Results

### Baseline demographic and clinical characteristics

Baseline demographic and clinical characteristics of the study group are described in Table 1. As expected, statistically significant differences in age, years of disease evolution, presence of obesity (p<0.001), ischemic heart disease (p<0.01), hypertension (p<0.001), dyslipidemia (p<0.001) and treatment (p<0.001) were observed between the two types of DM. Compared to patients with T1D, patients with T2D had significantly higher BMI (p<0.001) and age (p<0.001) and lower diabetes duration (p<0.001).

**Table 1:** Demographic and clinical characteristics of the study population.

Variable	T1D (n=53)	T2D (n=112)	p-Value
Age, years	41 (31–48)	60 (51–66)	<0.001
Duration, years	16 (12–18)	12 (7–18)	<0.001
Obesity, %	15.1	75.9	<0.001
Obesity type, %			
Low weight	3.8	0	<0.001
Normal weight	47.2	6.3	
Overweight	34	17.9	
Obesity type 1	13.2	41.1	
Obesity type 2	0	24.1	
Obesity type 3	1.9	10.7	
Comorbidities, %			
Microangiopathy	5.7	5.4	ns
Nephropathy	26.4	24.1	ns
Retinopathy	30.2	26.8	ns
Cerebrovascular accident	0	0.9	ns
Neuropathy	18.9	14.3	ns
Ischemic heart disease	0	11.6	0.01
Peripheral arterial disease	3.8	3.6	ns
Hypertension	9.4	59.8	<0.001
Dyslipemia	18.9	75	<0.001
Smokers, %	30.2	25	ns
Sun exposure, %			
Very low	2.3	8.6	ns
Sufficient	25	31.2	ns
High	72.7	60.2	ns
Alcohol, %	0	0.9	ns
Physical activity, %			
Sedentary	15.9	30.2	ns
Moderate	59.1	48.1	
Moderate active	18.2	17.9	
Active	6.8	3.8	
Previous osteoporotic fractures, %	20.8	30.4	ns
Family history of osteoporotic fracture, %	13.2	12.5	ns
Rheumatoid arthritis, %	1.9	3.6	ns
Medication, %			
Metformin	5.7	77.7	<0.001
DPP4 inhibitors	0.0	45.5	<0.001
Secretagogues	0.0	22.3	<0.001
Glitazones	0.0	11.6	<0.001
GLP1 agonists	1.9	26.8	<0.001
Insulin	94.3	64.3	<0.001

Data are presented as median (interquartile range [IQR]) for nonparametric variables. p-Value for difference between type 1 and type 2 diabetes patients. T1D, type 1 diabetes; T2D, type 2 diabetes; ns, non-significant.

No differences were found between both groups in gender, rheumatoid arthritis, smoking habit, alcohol intake, sun exposure, physical activity, personal or family history of previous fractures, as well as other comorbidities (microangiopathy, nephropathy, retinopathy, cerebrovascular accident, neuropathy and peripheral arterial disease).

### Bone mineral density

No statistically significant differences were found in BMD measured as femoral and lumbar T- and Z-scores between the two DM groups (Table 2).

**Table 2:** Bone mineral density and bone turnover markers in the two groups of diabetic.

Variable	T1D (n=53)	T2D (n=112)	p-Value
Hip T-score, SD	0.14 ± 1.0	0.14 ± 1.0	ns
Lumbar spine T-score, SD	0.28 ± 1.62	0.98 ± 1.74	ns
Hip Z-score, SD	0.71 ± 1.08	0.8 ± 1.43	ns

Normal bone, % (n)	79.2 (42)	73.1 (82)	ns
Low bone mass, % (n)	20.8 (11)	22.6 (25)	ns
Osteoporosis, % (n)	0 (0)	4.3 (5)	ns

HbA_1c_, %	7.92 ± 1.24	7.38 ± 1.27	0.022
Glucose, mg/dL	160.34 ± 81.9	144.99 ± 39.28	ns
PTH, pg/mL	25.52 ± 8.4	31.17 ± 18.15	ns
Vitamin D, ng/mL	21.71 ± 7.09	19.09 ± 6.74	0.029
Creatinine, mg/dL	0.81 ± 0.17	0.93 ± 0.41	0.016
Colesterol, mg/dL	190.43 ± 35.25	172.47 ± 32.08	0.001
Triglycerides, mg/dL	85.25 ± 44.27	152.12 ± 79.05	<0.001
OC, µg/L	17.65 ± 7.29	12.73 ± 6.23	<0.001
β-CTX, µg/L	0.31 ± 0.19	0.26 ± 0.18	ns
P1NP, µg/L	55.17 ± 37.25	37.52 ± 17.72	<0.001

Data are presented as mean ± SEM for continuous variables and as median (interquartile range [IQR]) for nonparametric variables. p-Value for difference between type 1 and type 2 diabetes patients. T1D, type 1 diabetes; T2D, type 2 diabetes; ns, non-significant.

We next classified patients according to the WHO criteria for the diagnosis of osteoporosis. Consequently, 42 patients (79.2 %) with T1D had normal BMD, 11 patients (20.8 %) had low bone mass and none of them had osteoporosis. In T2D, 82 patients (73.1 %) had normal BMD, 25 patients (22.6 %) had low bone mass and 5 (4.3 %) were osteoporotic. No statistically significant differences in osteoporosis or osteopenia prevalence were found between the two groups.

Since the frequency of osteoporosis was very low in both groups, we grouped patients with osteopenia and/or osteoporosis in one group (named bone pathology) and compared all clinical and biochemical parameters with patients with normal bone.

### Bone turnover markers

**Figure 1:**
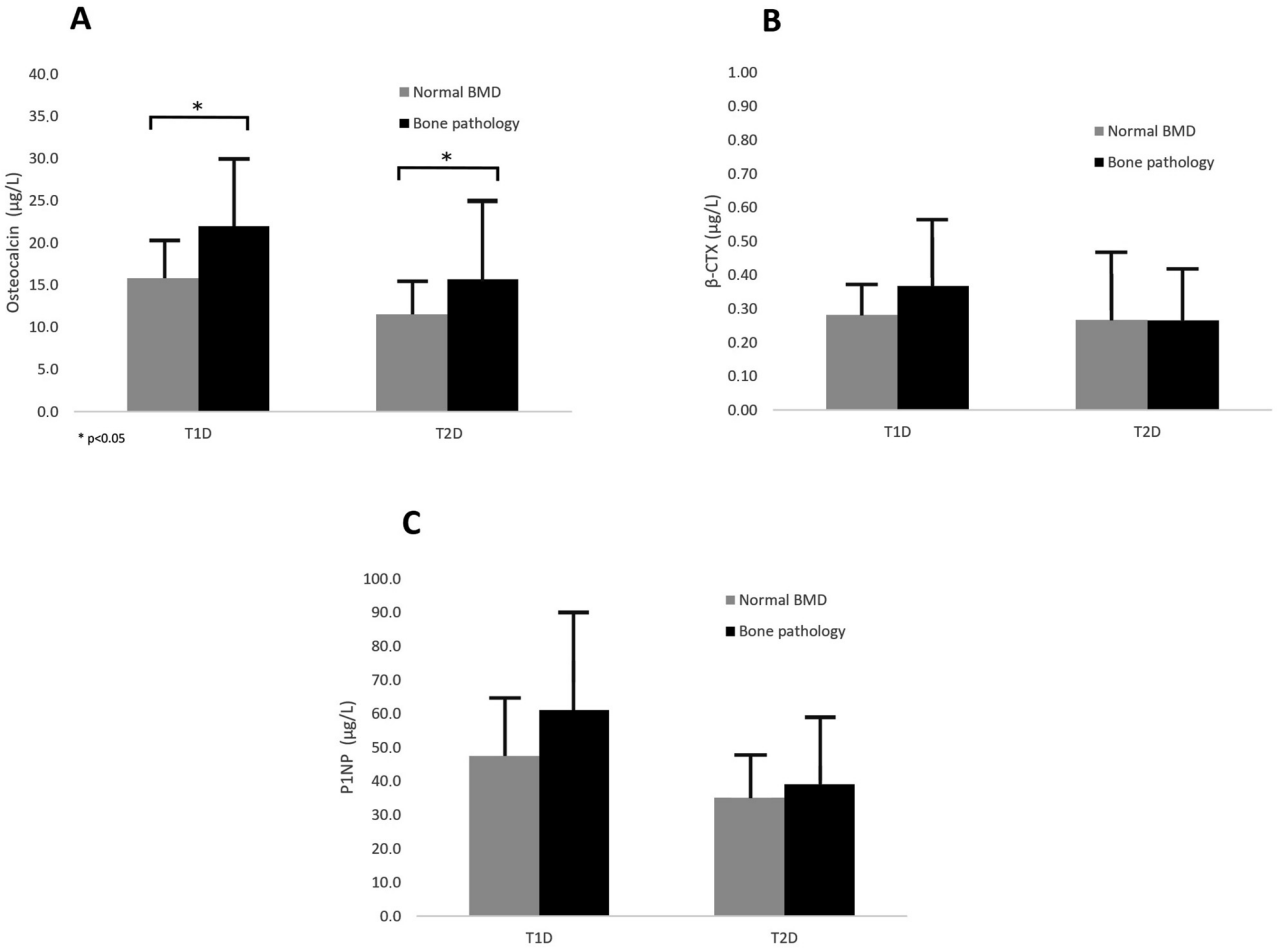
Graph shows levels of the bone turnover markers OC (A), β-CTX (B) and P1NP (C) in T1D and T2D in patients with normal bone mineral density compared to patients with bone pathology (osteopenia or osteoporosis) at the time of recruitment. Bars represent mean ± SEM. ^*^p-Values determine significant differences (p<0.05).

We compared BTM in patients presenting bone pathology with those with normal BMD. Both in T1D and T2D, significantly higher OC concentrations were detected in patients who had bone pathology compared with those with adequate BMD (T1D: 15.8 ± 5.1 vs. 22.0 ± 8.4 μg/L, p=0.03 and T2D: 11.5 ± 4.3 vs. 15.7 ± 9.6 μg/L, p=0.021). P1NP in T1D (47.5 ± 18.4 vs. 61.0 ± 30.3 μg/L) and T2D (35.1 ± 13.2 vs. 39.0 ± 20.4 μg/L) and β-CTX in T1D (0.28 ± 0.1 vs. 0.37 ± 0.2 μg/L) and T2D (0.27 ± 0.21 vs. 0.26 ± 0.16 μg/L) tended to be higher in patients with bone pathology although this change did not reach statistical significance (Figure 1).

Interestingly, significantly higher levels of the bone formation markers OC (p<0.001) and P1NP (p<0.001) were found in patients with T1D compared with T2D (Table 2). No statistically significant differences in β-CTX levels were found between the two groups. A significant number patients had OC levels below population reference values as at the time of enrolment, 28 (53 %) T1D and 91 (81 %) T2D.

The possible associations between BTM and other analytical and clinical markers were explored. Importantly, in patients with T1D a significant reverse correlation was found between levels of OC and BMD (hip Z-score: r=−0.509; p=0.016, hip T-Score: r=−0.399; p=0.021 and lumbar T-Score: r=−0.329; p=0.022). Also, in patients with T2D, levels of OC and β-CTX negatively correlated with hip T-score (r=−0.327; p=0.006 and r=−0.238; p=0.048, respectively).

Interestingly, significant correlations between OC plasma levels and glucose metabolism were found. In T1D patients OC significantly and inversely correlated with glycated haemoglobin levels (HbA_1c_%: r=−0.343; p=0.020). In T2D, all BTMs, negatively associated with fasting blood glucose (OC: r=−0.229; p=0.018, β-CTX: r=−0.283; p=0.003 and P1NP: r=−0.197; p=0.042) and β-CTX with HbA_1c_ (HbA_1c_%: r=−0.374; p<0.001).

We next evaluated the analytical performance of each BTM just as the cut-off values with the best discriminating power to detect bone pathology (Table 3). The areas under the curve (AUC) for β-CTX and P1NP to detect bone pathology were not significant neither in T1D nor in T2D. Multivariable analysis showed that OC independently associated with bone pathology (odds ratio [OR]: 1.16; CI: 1.05–1.28; p=0.003).

**Table 3:** Cut-off values and AUC of osteocalcin to detect bone pathology.

	Cut-off, µg/L	Sensitivity, %	Specificity, %	PPV, %	NPV, %	AUC	95 % CI	p-Value
T1D	20.54	67	86	67	86	0.732	0.52–0.94	0.038
T2D	12.88	65	79	57	84	0.697	0.57–0.80	0.007

T1D, type 1 diabetes; T2D, type 2 diabetes; PPV, positive predictive value; NPV, negative predictive value; AUC, area under the curve; CI, confidence interval.

### Vitamin D receptor gene polymorphisms

#### *VDR* allelic and genotypic frequencies

Frequencies of genotypes and alleles for the *VDR* gene polymorphisms in the whole population and in the subgroups (T1D and T2D) and the comparisons with the control population (the population European of 1000 Genomes Project and its subpopulation Iberian in Spain (IBS)) are shown in Table 4. The distribution of genotypes agreed with that expected according to Hardy–Weinberg equilibrium. The highest frequencies of heterozygous genotypes for all polymorphisms examined were recorded.

**Table 4:** Comparison of genotypic and alleles frequencies between the different study groups and controls.

SNP	Control population		All patients			T1D			T2D		
	EUR	IBS									
	GF	GF	GF	p-Value^a^	p-Value^b^	GF	p-Value^a^	p-Value^b^	GF	p-Value^a^	p-Value^b^
***BsmI* (rs1544410)**											
Genotype, G/A											
G/G	37	31	32.2	ns	ns	33.3	ns	ns	31.4	ns	ns
A/G	45	50	48.3			50			47.1		
A/A	18	19	19.5			16.7			21.6		
Allele											
G	60	56	53.5	ns	ns	54.3	ns	ns	53	ns	ns
A	40	44	46.5			45.7			47		
***ApaI* (rs7975232)**											
Genotype, C/A											
C/C	23	23	22	<0.001	<0.001	21.1	<0.001	<0.05	22.6	<0.001	<0.001
A/C	34	36	51.6			57.9			47.2		
A/A	43	39	26.4			21.1			30.2		
Allele											
C	45	43	48.6	ns	ns	50	ns	ns	47.5	ns	ns
A	55	57	51.4			50			52.6		
***TaqI* (rs731236)**											
Genotype, T/C											
T/T	38	33	34.4	ns	ns	35.9	ns	ns	33.3	ns	ns
T/C	44	48	48.9			51.3			47.1		
C/C	18	19	16.7			12.8			19.6		
Allele											
T	60	57	54.9	ns	ns	58.3	ns	ns	54.9	ns	ns
C	40	42	45.1			41.6			45.1		
***FokI* (rs2228570)**											
Genotype, C/T											
T/T	16	13	7.8	ns	ns	10	ns	ns	6	<0.05	ns
C/T	44	39	41.1			42.5			40		
C/C	40	48	51.1			47.5			54		
Allele											
T	38	33	34.7	ns	ns	36.8	ns	ns	36.8	ns	ns
C	62	67	65.4			63.2			63.2		

Data are presented as percentage. ^a^p-Value: for difference between data from control patients (population European of 1000 Genomes Project) and ^b^p-value: for difference between data from control patients (European sub-population [Iberian population in Spain]) with T1D and T2D patients. EUR, European; IBS, Iberian; GF, genotypic frequency; T1D, type 1 diabetes; T2D, type 2 diabetes; ns, non-significant.

No statistically significant differences were found in the genotypic and allele frequencies of the studied SNPs in T1D and T2D (except for *ApaI*). Thus, in the following polymorphisms were evaluated by grouping T1D and T2D into a single group. A significant association of the *ApaI* polymorphism with DM was found. A/C genotype was more frequent in patients with T1D (p<0.001) and in patients with T2D (p<0.001) compared to the control population. A significant association of the *FokI* polymorphism with T2D was also found when compared with the European population but not when comparing with the iberian subpopulation (Table 4).

#### Association of the *VDR* polymorphism with bone mineral density and bone turnover markers

##### BsmI

**Figure 2:**
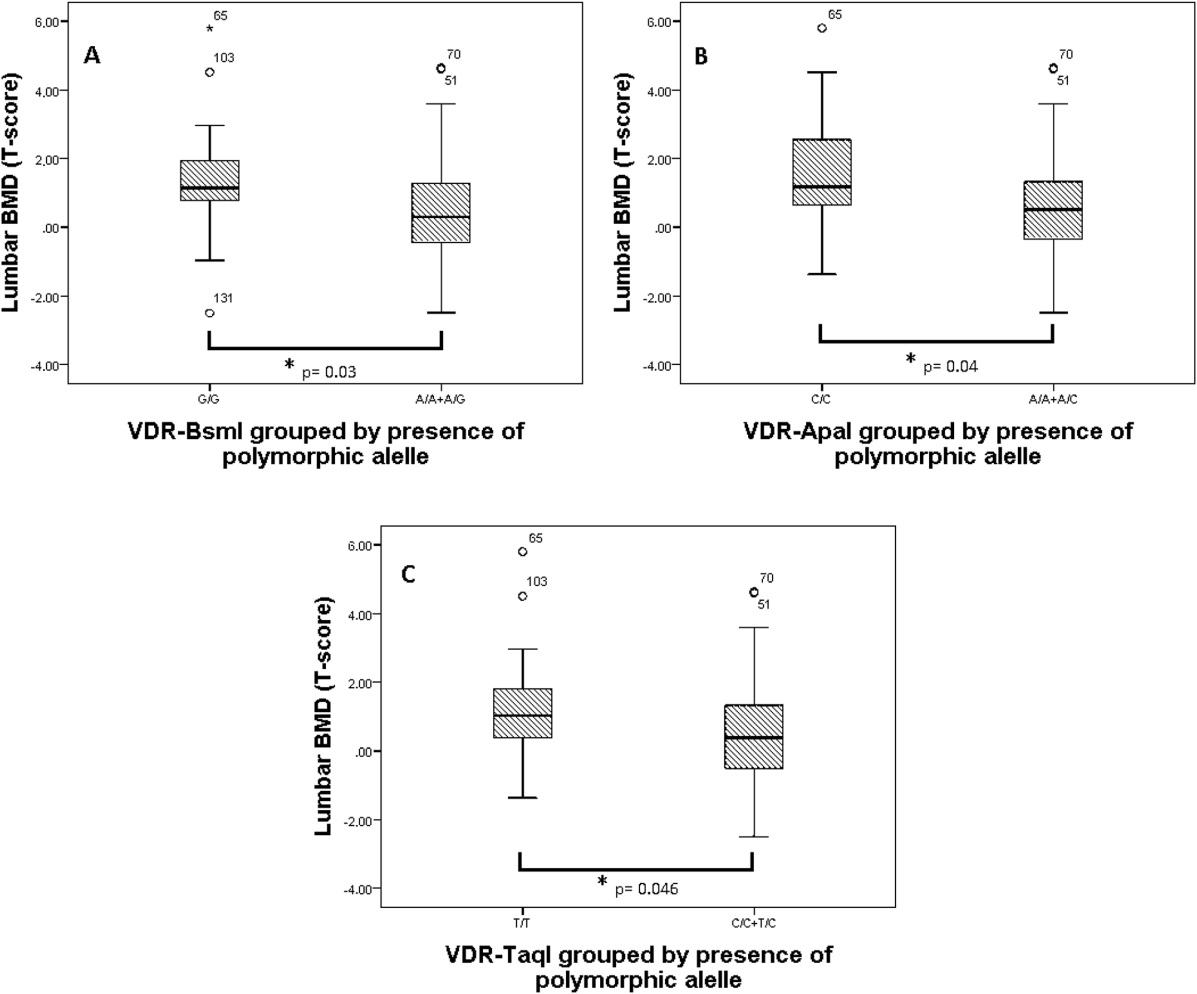
Graph shows levels of BMD (Lumbar T-score) according to *BsmI* (A), *ApaI* (B), and *TaqI* (C) SNPs in the *VDR* gene grouped by presence of polymorphic allele. Data are expressed as mean ± SEM. ^*^p-Values determine significant differences (p<0.05).

Significant lower lumbar BMD (T-score) was found in patients with the A allele (the recessive model (GG vs. A/A + A/G) (1.36 ± 1.16 vs. 0.50 ± 1.54; p=0.03)) (Figure 2). Similarly, BTM associated with the *BsmI* polymorphism. Concentrations of OC and β-CTX were significantly higher in carriers of the allele G vs. non-carriers (dominant model (G/G + G/A vs. A/A)), for OC (15.45 ± 7.98 vs. 12 ± 6.68 μg/L; p=0.044) and for β-CTX (0.31 ± 0.23 vs. 0.19 ± 0.09 μg/L; p=0.016). Similarly, the *BsmI* genotype also associated with β-CTX levels (G/G=0.28 ± 0.17 μg/L; A/G=0.34 ± 0.26 μg/L; A/A=0.19 ± 0.09 μg/L (p=0.027)).

##### ApaI

Lower lumbar BMD (T-score) was observed in presence of the A allele (the recessive model (CC vs. A/A + A/C) (1.53 ± 1.98 vs. 0.56 ± 1.44; p=0.04)) (Figure 2). This SNP also associated with OC levels, a statistically significant difference was observed in OC among the different genotypes (C/C=13.0 ± 6.04 μg/L; A/C=14.05 ± 5.02 μg/L; A/A=9.90 ± 3.21 μg/L (p=0.031)) and these concentrations were significantly higher in carriers of the allele C (dominant model (C/C + C/A vs. A/A)(13.73 ± 5.29 vs. 9.89 ± 3.21 μg/L; p=0.011).

##### TaqI

Patients with the allele C of the *Taql* (the recesive model (T/T vs. T/C + C/C) had significantly lower lumbar BMD (T-score) (1.27 + 1.54 vs. 0.49 ± 1.62; p=0.046)) (Figure 2). Likewise, this SNP associated with OC levels (T/T=14.05 ± 5.82 μg/L; T/C=12.86 ± 4.34 μg/L; C/C=8.97 ± 2.87 μg/L; (p=0.021)). Individuals with T allele (dominant model T/T + T/C vs. C/C) had significantly higher OC levels than those without the T allele (13.40 ± 5.05 vs. 8.97 ± 2.87 μg/L; p=0.006).

##### FokI

No associations were found between *FokI* and BMD but OC was significantly higher in patients with C allele (recessive model T/T vs. C/C + C/T (11.13 ± 0.89 vs. 15.18 ± 8.17 μg/L; p=0.004)).

Data of association of the *VDR* polymorphism with BMD and BTM are provided in the Supplemental Material (Supplemental Table 1).

## Discussion

The main findings of the current study are the following: (1) There was no difference in BMD between patients with T1D or T2D, but the formation markers OC and P1NP were lower in T2D compared to T1D; (2) a high percentage of diabetic patients had decreased levels of OC compared to reference values; (3) lower levels of OC associated with higher BMD and poorer glycemic control; (4) the alleles A of *BsmI*, A of *ApaI* and C of *TaqI* associated with lower lumbar BMD and (5) the alleles G of *BsmI*, C of *ApaI* and T of *TaqI* and C of F*okI* associate with higher OC levels.

### Bone mineral density in patients with type 1 and type 2 diabetes

In the present study we did not find differences in BMD nor in the prevalence of bone pathology between T1D and T2D patients despite the difference in age, an influential factor in the bone mass of an individual. These results are consistent with the study of Leidig–Bruckner et al. [15] where the prevalence of osteoporosis in both genders was equivalent in T1D and T2D, but lower in T2D compared to healthy population. The study by Díaz-Curiel et al. in a spanish cohort of healthy women found a prevalence of osteopenia (13 %) slightly lower than that found in our study for T1D of the same age [16]. This could be explained by the fact that T1D develops years before reaching peak bone mass and, therefore, the change in metabolism due to the presence of the disease affects bone development. In T2D we observed lower prevalence of osteopenia than expected for the same age range in the healthy population (22.6 % vs. 42–50 %) suggesting that the use of DEXA may not be the best tool to classify diabetic patients [16].

### Usefulness of bone turnover markers in diabetic patients

Different studies have evaluated the ability of BTM to predict the rate of bone loss, noting that higher levels of BTM associate with a greater rate of bone loss. Our results are consistent with previous work describing paradoxically, that OC levels are lower than the references range both in T1D and T2D, reflecting reduced bone formation in both types [9], [10], [11], [12].

Our study shows that serum levels of OC and P1NP were lower in patients with T2D than in patients with T1D without differences in serum levels of β-CTX between both groups. Also, these results are in accordance previous works [17], [18], [19], [20], [21]. Insulin has an anabolic effect on the bone, so the finding of lower OC in T2D could be related to the insulin resistance which is characteristic of these patients.

This study expands on these findings by demonstrating decrease OC associated with higher BMD and ROC curves analysis suggests that OC may be a useful screening tool to select diabetic patients with probable bone pathology. OC regulates bone formation but also has effects on other tissues, such as the pancreas and adipose tissue, where it is involved in glucose regulation and energy metabolism. OC is regulated by a number of hormones such as insulin which binds to osteoblasts causing the secretion of OC and which in turn promotes β-cell proliferation and increased insulin secretion thus contributing to osteometabolic control [22]. Our finding that OC levels significantly and negatively correlated with HbA_1c_ in T1D and with fasting blood glucose in T2D is consistent with this idea that OC functions as a hormone that regulates glucose metabolism [23]. The close relationship between OC and energy metabolism, dysregulated in DM, suggests the need for a lower OC cut-off point to screen for bone alterations in individuals with DM. In fact, we proposed a lower cut-off point for OC for diabetic patients which, due to its high NPV, could aid clinicians in identifying patients with low risk of suffering bone mass loss.

### Association of *VDR* gene polymorphisms with bone metabolism

The association of several SNPs in the *VDR* gene with the risk of osteoporosis was investigated.

Data show that *ApaI* was the only SNP associated with DM. These results are in agreement with other investigations that assessed the *VDR* polimorphism across different types of diabetes [24], [25], [26]. Analysis of the genotypic frequencies of *BsmI* and *FokI* obtained in our study are very similar to those obtained by Ji et al. [27] in a Caucasian population and in two spanish populations [28] which reported that the frequencies of *BsmI* and *FokI* genotypes do not differ between the control group and patients with DM1. However, other studies carried out in other ethnic groups show an association between *VDR* polymorphisms and DM [29, 30].

We found association between alleles of the polymorphisms in *BsmI*, *ApaI* and *TaqI* and lumbar BMD, as well as of all the studied polymorphisms with OC plasma levels. These results are in accordance with the meta-analysis of Thakkinstian et al. [31], of Jia et al. [32], one study in pediatric population [33] and those published by Álvarez-Hernández et al. [10] where the presence of the A/A genotype of *BsmI* vs. the combination of the other two genotypes showed lower lumbar BMD. This study concludes that the G/G of *BsmI*, C/C of *ApaI* and C/C of *TaqI* genotypes are somehow associated with higher lumbar BMD and with higher circulating OC. Our study is consistent with these findings, except for *TaqI* where our result is the opposite, lower lumbar BMD values and lower OC levels in the presence of the C allele of *TaqI*. These differences observed in *TaqI* may be due to the selected population, since there is great variability in the results of studies on the influence of *VDR* SNPs on BMD and BTM levels [13]. This could possibly be due to linkage disequilibrium, insufficient sample sizes or inhomogeneity of the populations studied, etc. In addition, most studies have been performed in pre- and postmenopausal women and there are few results in men. One strength of the present study is that we expand these previous results to a diabetic population that includes males.

Taken together these results suggest that the *BsmI*, *ApaI* and *TaqI* genotypes are potentially associated with BMD in diabetic patients, but are not specific to diabetic disease since the frequency distribution does not differ from the general population. Due to the negative effect of certain alleles on lumbar BMD and OC, this study suggests a role of *VDR* gene polymorphisms as potential contributors to bone loss in diabetic patients. However, future studies will have to elucidate the clinical utility.

### Limitations

Some limitations of the present study should be acknowledged. First, bone quality was not assessed by trabecular bone Score o qCT analysis. Also, sample size was somehow limited for a genetic study. Finally, this is a cross-sectional study and no follow-up of BTM was performed to evaluate the possible clinical utility of OC in patients monitoring.

## Conclusions

In conclusion, the data obtained in our study reveal that there is no difference in BMD between T1D and T2D patients and OC could be a candidate marker to screen for bone loss in diabetic patients. The high NPV of the cut-off point for OC suggests that OC could be useful in ruling out the risk suffering bone loss, allowing offering a personalized clinical approach to prevent this pathology. Future studies are needed to validate these new cut-off points associated with bone quality.

## Supplementary Material

Supplementary Material
