# Supplementary material for: Osteocalcin associates with bone mineral density and VDR gene polymorphisms in type 1 and type 2 diabetes
Source: Adv Lab Med. 2023 Oct 24;5(1):46–55. doi: 10.1515/almed-2023-0131 (PMC11019880; doi:10.1515/almed-2023-0131)
Supplement: Supplementary file 1 — Supplementary Material [file j_almed-2023-0131_suppl_001.docx]

**Supplemental Table 1.**Associations of the VDR gene polymorphisms with the BMD and BMT

| ***VDR/BsmI*** | **Genotype**  **GG** | **Genotype**  **AG** | **Genotype**  **AA** | ***p*** | **Allele G** | ***p*** | **Allele A** | ***p*** |
| --- | --- | --- | --- | --- | --- | --- | --- | --- |
| Hip T-score, SD | -0.01±1.06 | -0.22±1.09 | 0.27±1.72 | ns | -0.12±1.06 | ns | 0.06±1.32 | ns |
| Lumbar spine T-score, SD | 1.36±1.68 | 0.51±1.49 | 0.47±1.70 | ns | 0.83±1.60 | ns | 0.50±1.54 | 0.03 |
| Hip Z-score, SD | 0.33±0.45 | 0.72±1.10 | 0.47±1.15 | ns | 0.61±0.97 | ns | 0.64±1.09 | ns |
| OC (µg/L) | 14,41±6.91 | 16,21±8.69 | 12,00±6.68 | ns | 15.45±7.98 | 0.044 | 14.83±8.27 | ns |
| β-CTX (µg /L) | 0.28±0.17 | 0.34±0.26 | 0.19±0.09 | 0.027 | 0.31±0.23 | 0.016 | 0.30±0.23 | ns |
| P1NP (µg /L) | 42.62±24.52 | 48.29±37.00 | 34.58±19.63 | ns | 45.92±32.30 | ns | 43.86±32.88 | ns |
| ***VDR/ApaI*** | **Genotype**  **CC** | **Genotype**  **AC** | **Genotype**  **AA** | ***p*** | **Allele C** | ***p*** | **Allele A** | ***p*** |
| Hip T-score, SD | 0.55±1.55 | -0.28±1.05 | 0.07±1.24 | ns | -0.18±1.09 | ns | 0.38±1.25 | ns |
| Lumbar spine T-score, SD | 0.44±1.71 | 0.62±1.30 | 1.53±1.98 | ns | 0.85±1.53 | ns | 0.56±1.43 | 0.04 |
| Hip Z-score, SD | 0.41±1.14 | 0.67±1.07 | 0.59±0.40 | ns | 0.65±0.97 | ns | 0.56±1.08 | ns |
| OC (µg/L) | 13.0±6.04 | 14.05±5.02 | 9.90±3.21 | 0.031 | 13.73±5.29 | 0.011 | 12.95±4.86 | ns |
| β-CTX (µg /L) | 0.25±0.16 | 0.32±0.26 | 0.25±0.16 | ns | 0.29±0.22 | ns | 0.29±0.23 | ns |
| P1NP (µg /L) | 39.00±20.96 | 47.84±36.82 | 37.59±16.77 | ns | 44.63±32.14 | ns | 44.89±32.50 | ns |
| ***VDR/TaqI*** | **Genotype**  **TT** | **Genotype**  **TC** | **Genotype**  **CC** | ***p*** | **Allele T** | ***p*** | **Allele C** | ***p*** |
| Hip T-score, SD | 0.01±0.98 | -0.32±1.17 | 0.65±1.73 | ns | -0.17±1.09 | ns | -0.07±1.36 | ns |
| Lumbar spine T-score, SD | 1.27±1.54 | 0.37±1.56 | 0.82±1.78 | ns | 0.73±1.60 | ns | 0.49±1.61 | 0.046 |
| Hip Z-score, SD | 0.43±0.47 | 0.78±1.13 | 0.27±1.10 | ns | 0.67±0.98 | ns | 0.63±1.11 | ns |
| OC (µg/L) | 14.05±5.82 | 12.86±4.34 | 8.97±2.87 | 0.021 | 13.40±5.05 | 0.006 | 11.78±4.32 | ns |
| β-CTX (µg /L) | 0.28±0.17 | 0.32±0.26 | 0.20±0.10 | ns | 0.30±0.22 | ns | 0.28±0.23 | ns |
| P1NP (µg /L) | 43.3±24.2 | 46.6±36.5 | 36.9±22.1 | ns | 45.11±31.61 | ns | 44.06±33.48 | ns |
| ***VDR/FokI*** | **Genotype**  **TT** | **Genotype**  **CT** | **Genotype**  **CC** | ***p*** | **Allele T** | ***p*** | **Allele C** | ***p*** |
| Hip T-score. SD | -0,34±1.19 | 0,04±1.24 | 0,89±1.99 | ns | 0.10±1.27 | ns | -0.14±1.21 | ns |
| Lumbar spine T-score. SD | 0.72±1.77 | 0.51±1.44 | 1.38±1.17 | ns | 0.58±1.42 | ns | 0.62±1.61 | ns |
| Hip Z-score. SD | 0.46±0.90 | 0.58±1.19 | 1.77±0.10 | ns | 0.69±1.18 | ns | 0.51±1.00 | ns |
| OC (µg/L) | 15.92±8.95 | 14.32±7.18 | 11.13±0.89 | ns | 13.79±8.94 | ns | 15.18±8.17 | 0.004 |
| β-CTX (µg /L) | 0.32±0.26 | 0.26±0.15 | 0.17±0.08 | ns | 0.24±0.14 | ns | 0.29±0.21 | ns |
| P1NP (µg /L) | 47.94±38.28 | 40.24±22.71 | 35.61±5.95 | ns | 39.49±20.92 | ns | 44.38±32.08 | ns |

Note: Data are presented as mean ± SEM

p-value for difference between different genotypes and alleles

ns: non-significant.
